# Supplementary material for: Photoacoustic 2D actuator via femtosecond pulsed laser action on van der Waals interfaces
Source: Nat Commun. 2023 Apr 14;14:2135. doi: 10.1038/s41467-023-37763-8 (PMC10104871; doi:10.1038/s41467-023-37763-8)
Supplement: Supplementary file 1 — Supplementary Information [file 41467_2023_37763_MOESM1_ESM.pdf]

Supplementary Information for

Photoacoustic 2D actuator via femtosecond pulsed laser action on van der Waals interfaces

Xin Chen,[1,2] *Ivan M. Kislyakov,*[*,1] *Tiejun Wang,*[3] *Yafeng Xie,*[1,2] *Yan Wang,*[1,2] *Long Zhang,*[1,4] *and Jun Wang*[*,1,2,3,4]

[1]Photonic Integrated Circuits Center, Key Laboratory of Materials for High-Power Laser, Shanghai Institute of Optics and Fine Mechanics, Chinese Academy of Sciences, Shanghai 201800, China

[2]Center of Materials Science and Optoelectronics Engineering, University of Chinese Academy of Sciences, Beijing 100049, China

[3]State Key Laboratory of High Field Laser Physics, Shanghai Institute of Optics and Fine Mechanics, Chinese Academy of Sciences, Shanghai 201800, China

[4]Center for Excellence in Ultra-intense Laser Science, Chinese Academy of Sciences, Shanghai 201800, China

[*]Email: jwang@siom.ac.cn; iv.kis@siom.ac.cn

Supplementary Note 1. Optical drive system.

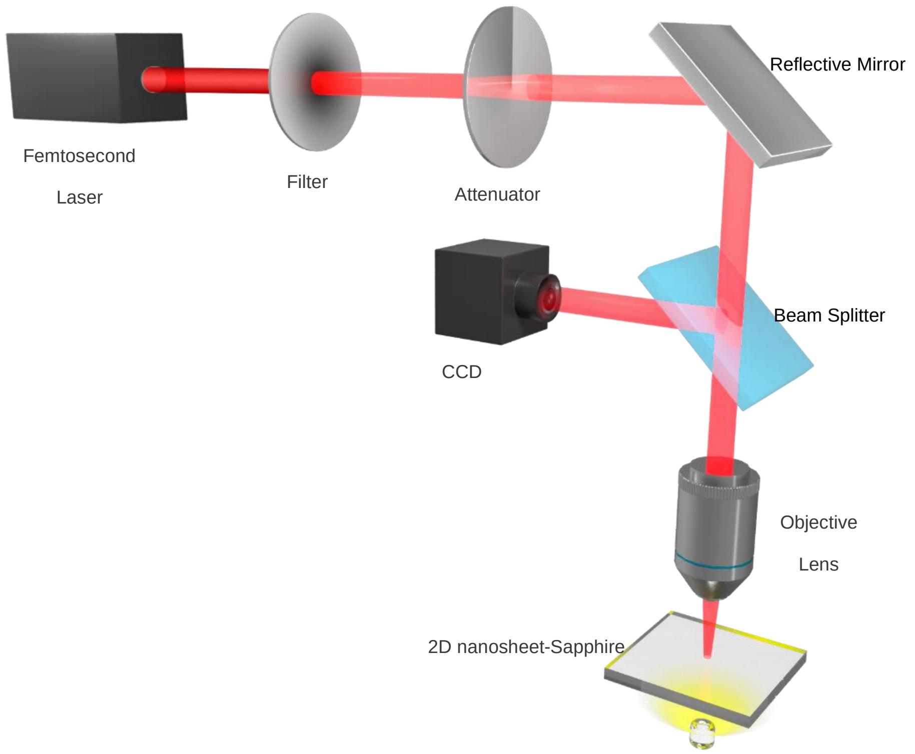

Supplementary Figure 1 The setup of our optical drive system.

Supplementary Note 2. Raman characterization

The Raman spectra from 100 to 300 cm^{-1} under 532 nm excitation is presented in Supplementary Fig. 2. The E_g mode at 142 cm^{-1} and A_{1g} mode at 210 cm^{-1} correspond to the out-of-plane and in-plane vibrations of selenium atoms, respectively, which demonstrates that the VSe_2 nanosheet owns 1T phase. It can be seen that the peak positions of Raman signals before and after laser irradiation are the same.

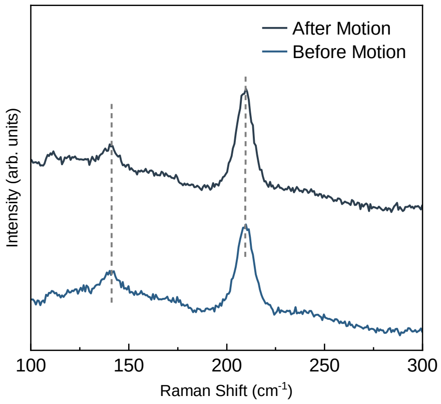

Supplementary Figure 2 Raman spectra of VSe_2 nanosheet before and after laser irradiation.

Supplementary Note 3. Roughness characterizations of the substrates by AFM

We have to mention that the fluctuation of the substrate in Fig. 1d is from the noise of AFM instrument. We used a more professional AFM instrument to characterize the roughness of our substrates, including sapphire, quartz, and silicon, as shown in Supplementary Fig. 3. The roughness is 0.0842 nm, 0.372 nm, and 0.202 nm, respectively. This data is also guaranteed by its manufacturer's product certificate.

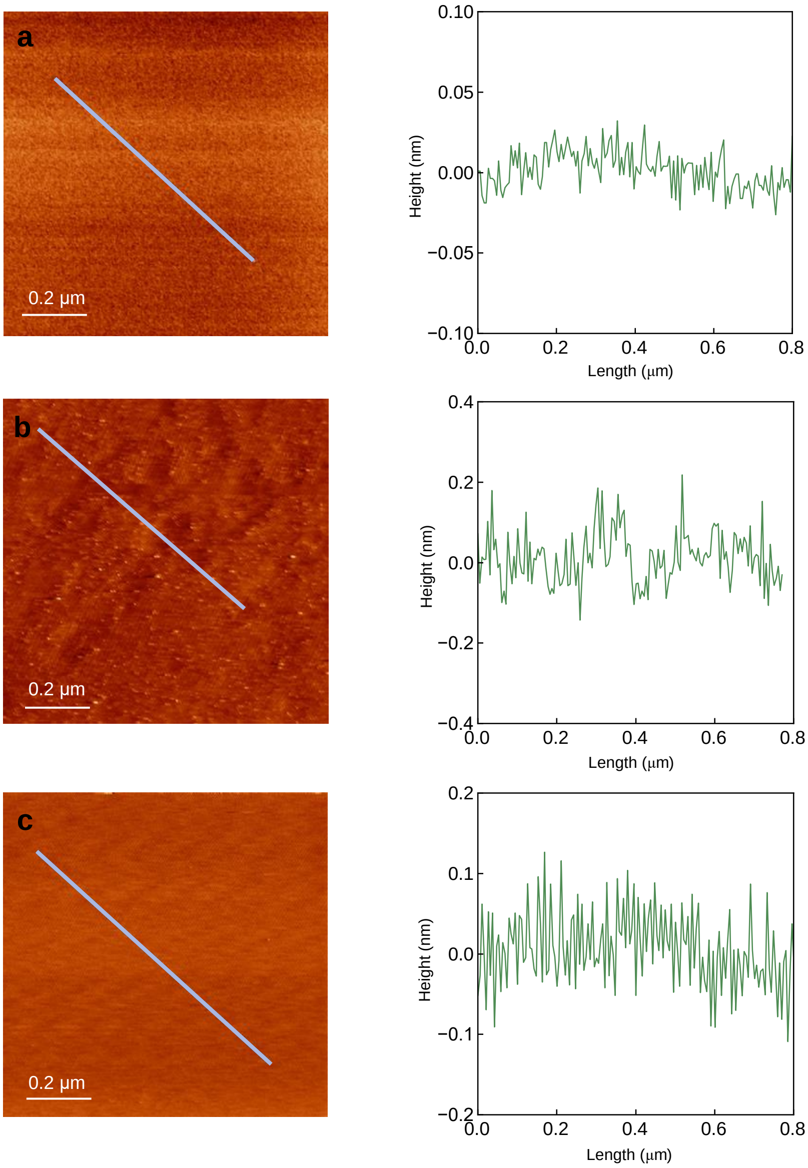

Supplementary Figure 3 Atomic Force microscopy characterizations of substrates. **a** Sapphire, **b** Quartz, and **c** Silicon.

Supplementary Note 4. Morphology of the VSe_2-sapphire contact

The general view of a transversal cut of a nanosheet is shown in Supplementary Fig. 4a. Along the length of the nanosheet, the contact is not uniform: extended regions of different length are found along its edges (red and green squares), in which, when zooming (Supplementary Figs. 4b and 4c), gaps without a substance (apparently filled with air) are observed. Subsequent zooming (Supplementary Figs. 4e and 4f) shows the gap thickness of ~10 nm and even more.

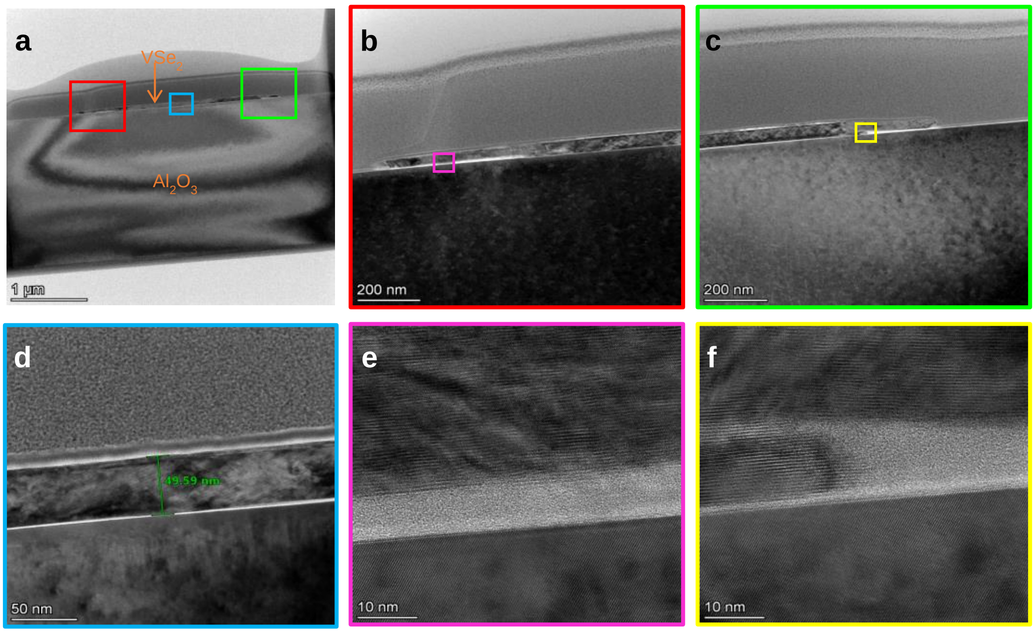

Supplementary Figure 4 Cross-sectional transmission electron microscopy images of a ME VSe_2 nanosheet on sapphire substrate. **a** General view of the contact. **b, c, d** Enlarged images of the red, green, and blue squares in **a**. The nanosheet thickness is about 50 nm. **e, f** Enlarged images of the pink square in **b** and the yellow square in **c**.

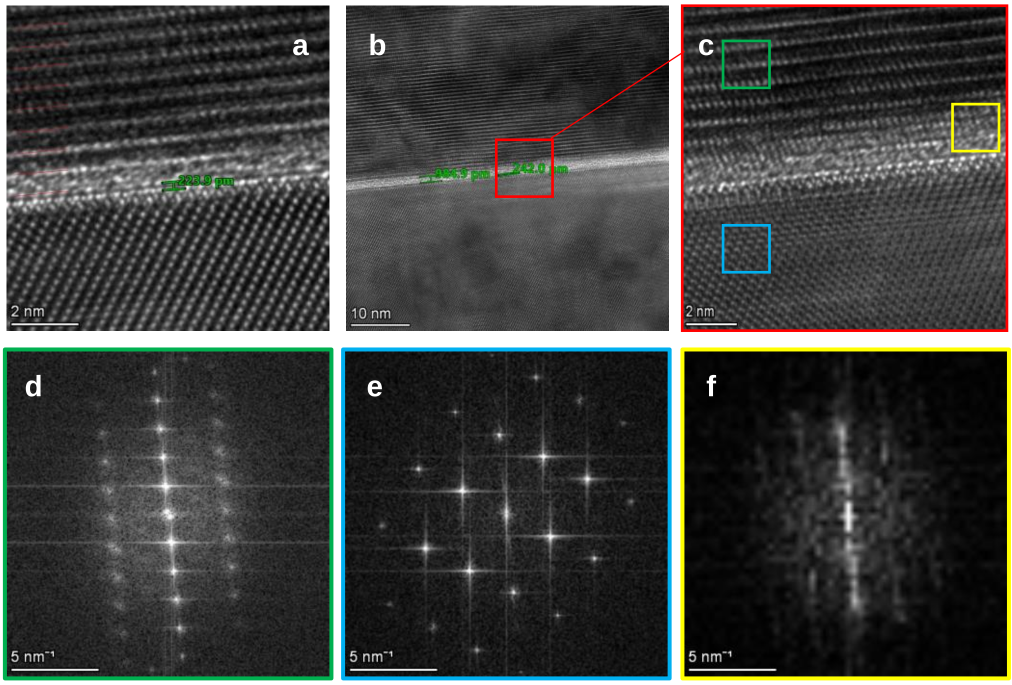

Supplementary Figure 5 Zoomed images of close contact area and FFT patterns.

The central part of the nanosheet demonstrates a close contact. The red lines in Supplementary Fig. 5a mark the VSe_2 layers, and the spacing between contacting layer and the substrate of 0.224 nm is clearly distinguishable (the uncertainty is 10-15%). It is in agreement with similar observation of the MBE VSe_2-sapphire contact by other authors [S1] and with our estimates of the contact distance by the hard-sphere model (0.255 nm).

Several layers on the contacting plane (~1 nm thickness in Supplementary Fig. 5b) are in a large extent defected, its crystal structure is partially smoothed out and therefore looks pale on a large scale. However, as it is seen at zooming (Supplementary Fig. 5c), the VSe_2 layers are still present, and the interlayer distance is not much affected. FFT patterns of regular VSe_2 layers and sapphire are shown in Supplementary Figs. 5d and 5e, respectively. The FFT of the defected area (Supplementary Fig. 5f) still reveals the residual VSe_2 identity.

Supplementary Note 5. The calculations in adhesion work

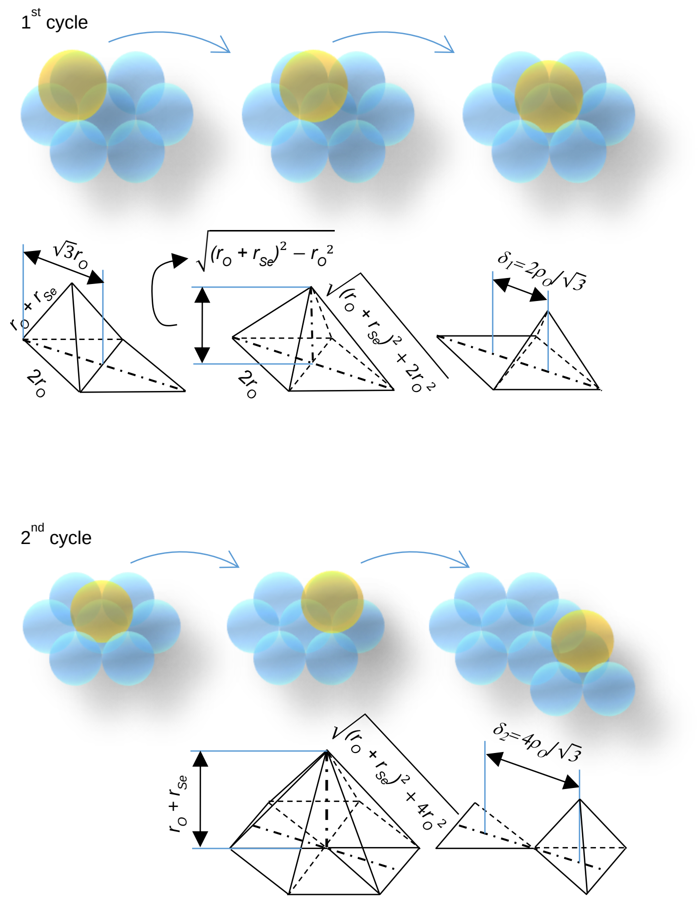

Supplementary Figure 6 Movement of the selenium atoms along the contact oxygen lattice of sapphire. The yellow and blue spheres represent selenium atoms and oxygen atoms. r_O and r_{Se} are the hard sphere radii of the surface atoms (oxygen from the sapphire side and selenium from VSe_2 side).

A selenium atom has the minimum energy lying at the center of the oxygen triangle (the first position in Supplementary Fig. 6):[S2]

$$E_{low} = -\frac{3C}{(r_O+r_{Se})^6}, \tag{S1}$$

where C is the vdW constant, r_O and r_{Se} are the radii of Oxygen and Selenium atoms. The transition of the selenium atom from one equilibrium position in the center of the oxygen triangle to another will be an alternation of two cycles: in the first cycle, the atom passes

through the center of the side of the triangle, and in the second through its vertex. Accordingly, its energy in the top position will be:

$$E_{up1} = -\frac{2C}{(r_O+r_{Se})^6}\{1+\frac{1}{[1+2(\frac{r_O}{r_O+r_{Se}})^2]^3}\} \quad \text{(S2)}$$

in the first cycle, and

$$E_{up2} = -\frac{C}{(r_O+r_{Se})^6}\{1+\frac{6}{[1+(\frac{2r_O}{r_O+r_{Se}})^2]^3}\} \quad \text{(S3)}$$

in the second cycle.

The relative change of adhesion energy is equal to the relative change in the energy of one atom:

$$\frac{\Delta W_1}{W_{adh}} = \frac{E_{up1}-E_{low}}{E_{low}} = \frac{2}{3[1+2(\frac{r_O}{r_O+r_{Se}})^2]^3} - \frac{1}{3}, \quad \text{(S4)}$$

$$\frac{\Delta W_2}{W_{adh}} = \frac{E_{up2}-E_{low}}{E_{low}} = \frac{2}{[1+(\frac{2r_O}{r_O+r_{Se}})^2]^3} - \frac{2}{3}. \quad \text{(S5)}$$

With the hard sphere radii obtained as the shortest half-distances between neighboring atoms laying in the contact planes, $r_O = 0.126$ nm [S3] and $r_{Se} = a_{lat}/2 = 0.168$ nm (VSe_2),[S4] Eq. (S4) gives $\Delta W_1/W_{adh} = -0.07255$, and Eq. (S5) gives $\Delta W_2/W_{adh} = -0.28352$. The total change over two cycles will be: $(\Delta W_1+\Delta W_2)/W_{adh} = -0.3561$. The total path over two cycles (according the scheme in Supplementary Fig. 6): $\delta_{tot} = \delta_1 + \delta_2 = 2\sqrt{3}r_O$.

Supplementary Note 6. Evaluation of the gradient optical forces acting on a VSe_2 nanosheet

The fundamental theory points out ponderomotive nature of the transversal force acting from electromagnetic radiation on a continuous material medium.[S5] The general description of the ponderomotive force density exerted in a bulk material with permittivity ε and mass density ρ in the electric field E is:[S6]

$$\boldsymbol{f}_{pm} = \rho_e \boldsymbol{E} + \varepsilon_0 \nabla \left(E^2 \rho \frac{\partial \varepsilon}{\partial \rho} \right) - \varepsilon_0 E^2 \nabla \varepsilon. \tag{S6}$$

Here the first term is due to free charges with density ρ_e. It turns to be zero with averaging over the light wave period. The second and third terms are dielectric ones. The Clausius-Mossotti equation gives:

$$\rho \frac{\partial \varepsilon}{\partial \rho} = \frac{(\varepsilon - 1)(\varepsilon + 2)}{3}, \tag{S7}$$

then

$$\varepsilon_0 \nabla \left(E^2 \rho \frac{\partial \varepsilon}{\partial \rho} \right) = \frac{\varepsilon_0 (\varepsilon - 1)(\varepsilon + 2)}{3} \nabla E^2 + \frac{\varepsilon_0 (2\varepsilon + 1)}{3} E^2 \nabla \varepsilon. \tag{S8}$$

Since we consider the nanosheet's material uniform in its plane, $\nabla_x \varepsilon = \nabla_y \varepsilon = 0$. At that, thermal expansion cannot disturb it during the field application time (380 fs). Therefore, using $E^2 = 2I/\varepsilon_0 c$ (here we put refractive index of the air $n = 1$), we get:

$$\boldsymbol{f}_{pm} = \frac{2(\varepsilon - 1)(\varepsilon + 2)}{3c} \nabla I, \tag{S9}$$

where ∇I is the light intensity gradient. The full force $\boldsymbol{F}_{pm}$ can be obtained by integrating of $\boldsymbol{f}_{pm}$ over the nanosheet's volume. The intensity gradient is nonzero only in the region of the edge of the laser spot, in the streak, the area of which we estimated as $a \times a_x$ with $a = 3.7$ μm and $a_x = 1$ μm. Therefore, the gradient force

$$F_{grad} = |\boldsymbol{F}_{pm}| = \frac{2(\varepsilon - 1)(\varepsilon + 2)}{3c} a a_x L \nabla_x I, \tag{S10}$$

where L is the nanosheet's thickness. Using an experimental value $\varepsilon(1040 \text{ nm}) \approx +1.6$,[S7] we get $F_{grad} = 178$ nN for the upper bound of $\nabla_x I = 1 \times 10^{21}$ W/m^3.

Theoretically, a permittivity gradient can arise due to ultrafast refraction nonlinearities like optical Kerr effect or free-carrier absorption. In this case the corresponding terms in Eq. (S6):

$$\boldsymbol{f}_{pm}^{(\nabla\varepsilon)} = \frac{2\varepsilon_0(\varepsilon-1)}{3} E^2 \nabla\varepsilon \tag{S11}$$

may be important. VSe_2 nanosheets are only known by certain saturable absorption,[S8,S9] which gives no impact into the optical force. However, an ultrafast nonlinear refraction index for another 2D semimetal, $ZrTe_2$, has been reported: $n_2 = 4 \times 10^{-20}$ m^2/W. [S10] At the typical intensity in our experiment of $I = 4 \times 10^{14}$ W/m^2, it can induce $\Delta n = 1.6 \times 10^{-5}$, which at the length a_x would result in $\nabla_x \varepsilon = 40.5$ m^{-1}. With these estimates, $F_{pm}^{(\nabla\varepsilon)} = f_{pm}^{(\nabla\varepsilon)} a^2 L = 6$ pN, which is negligible even the nonlinearity would turn out several orders of magnitude larger.

Supplementary Note 7. Optical, thermal, and acoustic properties of 2D materials and substrates

The optical, thermal, and acoustic properties of 2D materials (h-BN, MoS_2, WSe_2, and $PdSe_2$, $TiSe_2$, and VSe_2) and substrates (sapphire, quartz, and silicon) are collected. They are listed as follows:

Supplementary Table 1 Absorption coefficient ($\alpha_{E\perp c}$), reflectivity ($R_{E\perp c}$), linear thermal expansion coefficient (α_{TE}), and thermal conductivity (κ).

**Material**	**$\alpha_{E\perp c}$ (cm^{-1}) at 1040 nm**	**$R_{E\perp c}$ at 1040 nm**	**α_{TE} (10^{-6} K^{-1})**	**κ ($W\cdot m^{-1}\cdot K^{-1}$)**
**2D materials***				
**h-BN**	<9.5 [S11]	nonissue	38.0 (*c*-axis) [S12]<br>2.73 (*a*-axis) [S12]	2 (*c*-axis) [S13]<br>400 (*a*-axis) [S13]
**MoS_2**	4.8×10^3 [S14]	0.40 [S14]	8.65 (*c*-axis) [S15]<br>1.9 (*a*-axis) [S15]	2.0 (*c*-axis) [S16]<br>85 (*a*-axis) [S16]
**WSe_2**	4.0×10^4 (5L) [S17]	0.41 [S17]	16.7 (*c*-axis) [S15]<br>11.1 (*a*-axis) [S15]	1.5 (*c*-axis) [S18]
**$PdSe_2$**	2.0×10^4 (7L) [S19]	0.37 [S19]	no data found	1.51 (*c*-axis) [S20]<br>28.5 (12L, *a*-axis) [S21]
**$TiSe_2$**	**3.3×10^5** (exp.) [S22]<br>**5.8×10^5** (theory) [S23]	0.47 [S24]	19.2 (*c*-axis) [S25]<br>**18.4** (*a*-axis) [S25]	1.85 (*c*-axis) [S26]<br>≈20 (*a*-axis) [S27]
**VSe_2**	**3.3×10^5** (exp.) [S28]<br>**7.8×10^5** (theory) [S29]	0.48 [S28]<br>0.40 [S29]	9.8 (*c*-axis) [S25]<br>**18.6** (*a*-axis) [S25]	2.99 (*c*-axis) [S30]<br>7.32 (*a*-axis) [S30]
**substrates**				
**α-Al_2O_3 (sapphire)**	~0	nonissue	7.3 (*c*-axis) [S31]<br>8.1 (*a*-axis) [S31]	25.8 (*c*-axis) [S32]<br>23.0 (*a*-axis) [S32]
**JGS3 quartz glass [S33]**	~0		0.55	1.4
**Silicon**	21.7 [S34]		3.6 [S31]	148 [S35]

* The values characterize the bulk substance, unless the number of layers is specified.

Supplementary Table 2 Specific heat (C_p), density (ρ), average (upper bounds polycrystals) values of bulk modulus ($\bar{B}$), Young's modulus ($\bar{E}$), and Poisson's ratio ($\bar{\nu}$).

Material	C_p ($J \cdot K^{-1} \cdot kg^{-1}$)	ρ ($g \cdot cm^{-3}$)	$\bar{B}$ [S4] (GPa)	$\bar{E}$ [S4] (GPa)	$\bar{\nu}$ [S4]
**2D materials**					
**h-BN**	794 [S35]	2.18 [S35]	196.7 (mp-629015)	344.1	0.21
**MoS_2**	397 [S35]	5.06 [S35]	49.6 (mp-2815)	83.3	0.22
**WSe_2**	212 [S36]	9.2 [S35]	41.8 (mp-1821)	76.6	0.19
**$PdSe_2$**	266 [S20]	5.94 [S4]	36.6 (mp-2418)	60.1	0.23
**$TiSe_2$**	345 [S37]	4.69 [S4]	27.0 (mp-2194)	46.6	0.21
**VSe_2**	369 [S38]	5.825 [S39]	27.1 (mp-694)	50.4	0.19
**substrates**					
**α-Al_2O_3 (sapphire)**	775 [S35]	3.97 [S35]	231.8 (mp-1143)	368.7	0.23
**JGS3 quartz glass [S33]**	670	2.33	37 [S33]	72 [S33]	0.17 [S33]
**Silicon**	712 [S35]	2.33 [S35]	83.3 (mp-149)	151.3	0.20

Supplementary Note 8. Ultrafast carrier dynamics process of VSe_2 nanosheets

Ultrafast pump-probe technique is an informative technique to characterize electron relaxation processes[S40] and to study coherent acoustic phonons[S41]. Supplementary Fig. 7 shows the home-built microscopic pump-probe technique. The pump and probe beams can be selectively doubled to 520 nm.

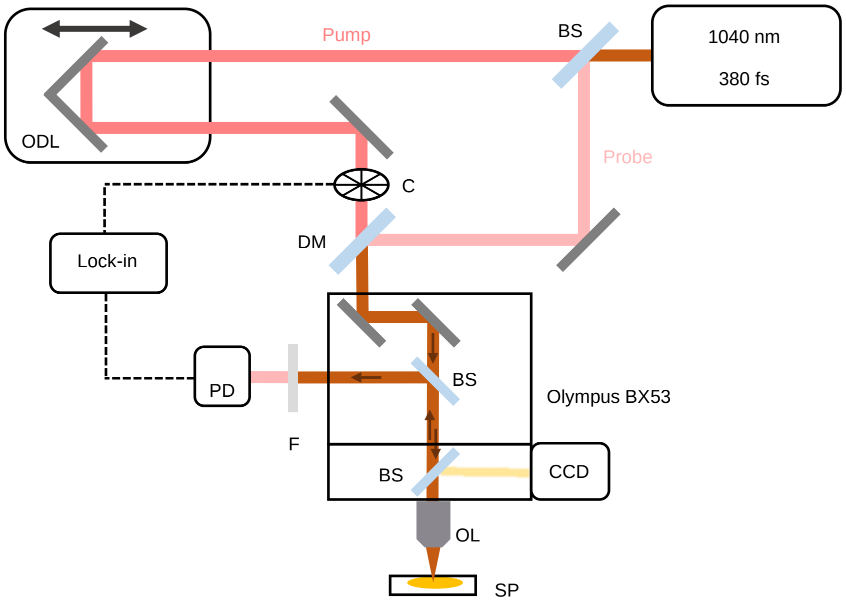

Supplementary Figure 7 The setup of the pump-probe system. BS: beam splitter; ODL: optical delay line; C: chopper; DM: dichroic mirror; OL: objective lens; SP: sample; F: filter; PD: photodiode.

Here we obtained time-resolved differential reflectivity signals of VSe_2 nanosheets on sapphire by pumping with both visible (520 nm) and infrared (1040 nm) fs laser beams, as shown in Supplementary Fig. 8.

When pumped at a wavelength of 520 nm, the transient reflection relaxes generally faster than when pumped at 1040 nm. The relaxation process can be well approximated by a bi-exponential model. The fitted fast and slow relaxation lifetime is τ_1 = 85.1 ps and τ_2 = 599.5 ps with the amplitudes A_1 = 0.516 and A_2 = 0.944. The Fourier spectrum

of the remainder, in addition to high-frequency noise, often detects the manifestation of coherent phonons with a frequency of 14.0 ± 0.2 GHz, and sometimes the presence of lower-frequency components in the region of 1 GHz.

When pumping at 1040 nm, we do not observe pronounced oscillations in the studied frequency range. This difference is explained by the fact that the photon energy of 520 nm (2.38 eV) exceeds the interband transition edge of VSe_2 (1.5 eV),[S40] absorption leads to the formation of new electron-hole pairs giving rise to a deformation photoacoustic effect, which is the most powerful mechanism of the excited coherent phonons.[S41] When the low-energy photon energy of 1040 nm (1.19 eV) is absorbed, the excitation of charge carriers inside the band does not induce coherent phonons, which is consistent with others.[S40]

The relaxation lifetime in Supplementary Fig. 6b is $\tau_1 = 402.2$ ps and $\tau_2 = 4148$ ps with the amplitudes $A_1 = 0.512$ and $A_2 = 0.907$. This is remarkably slower than the corresponding lifetime of the reported monolayer VSe_2, $\tau_1 = 16$ ps and $\tau_2 = 200$ ps, in the similar conditions of the intraband pumping (1.5 eV).[S40] It can be assumed that a significant decrease of the number of layers accelerates electron-phonon relaxation, which is also manifested in a large spread of the characteristic decay times in our experiments.

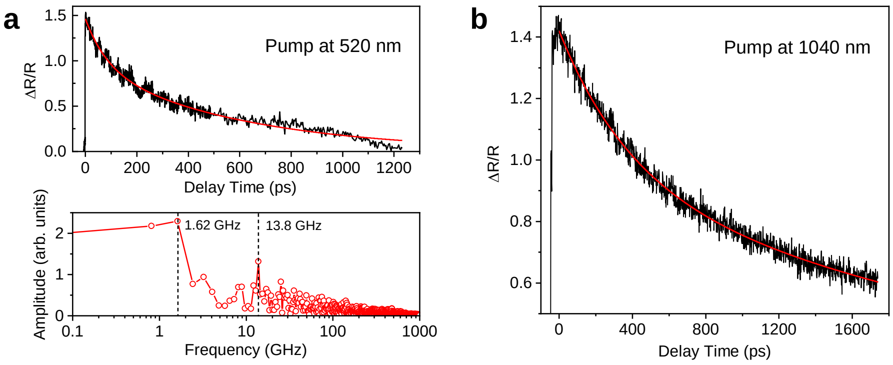

Supplementary Figure 8 Ultrafast dynamics processes of VSe_2 nanosheets on sapphire. **a** Pumping at 520 nm. A bi-exponential fitting curve is shown by the red curve. The lowers graph represents a Fourier spectrum of the difference between the experimental and fitting signals. **b** Pumping at 1040 nm. The black and red lines are the experimental signal and its fitting curve.

Supplementary Note 9. AFM characterizations of VSe_2 and $TiSe_2$ nanosheets

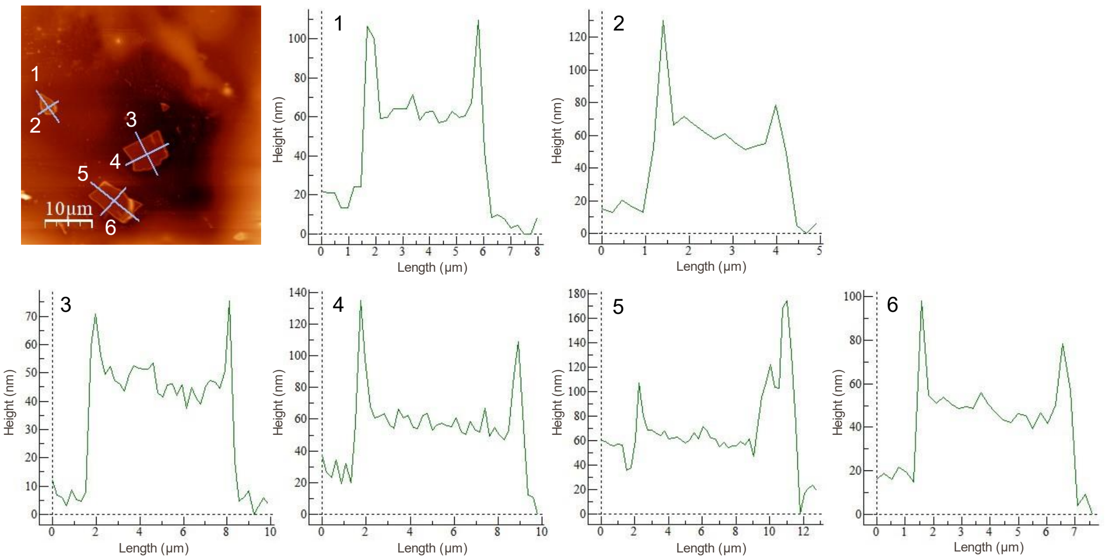

Supplementary Figure 9 Height profiles of VSe_2 nanosheets.

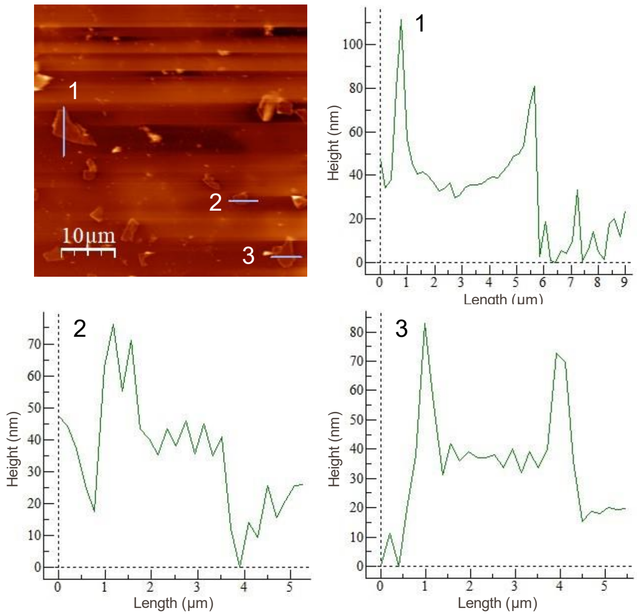

Supplementary Figure 10 Height profiles of VSe_2 nanosheets.

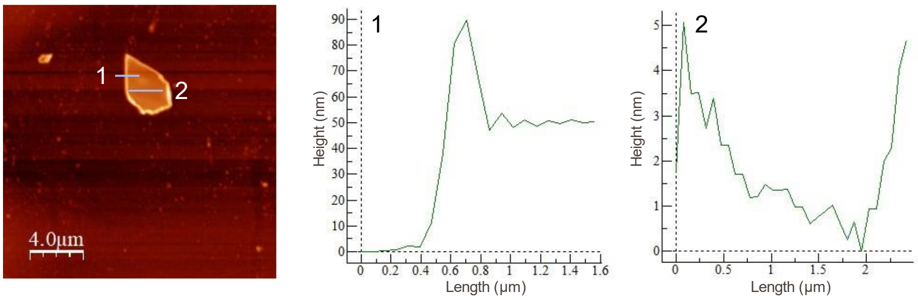

Supplementary Figure 11 Height profiles of one VSe_2 nanosheet.

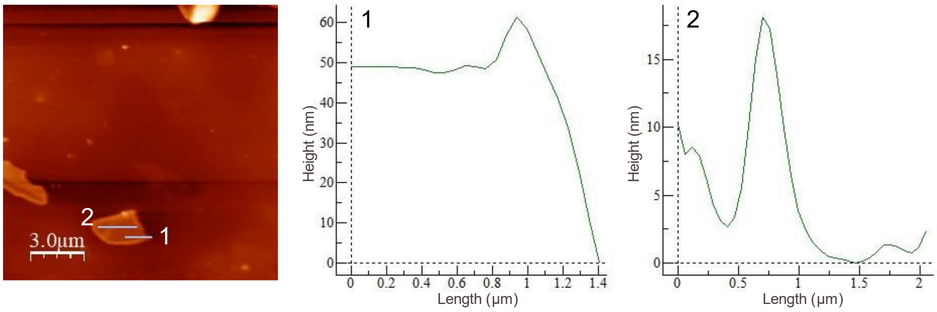

Supplementary Figure 12 Height profiles of one VSe_2 nanosheet.

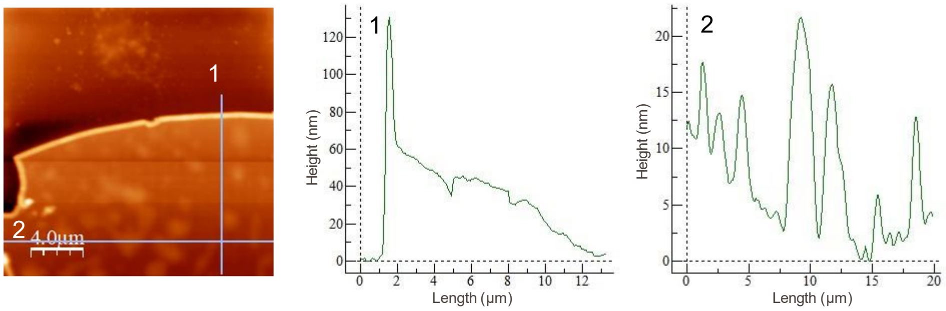

Supplementary Figure 13 Height profiles of one VSe_2 nanosheet.

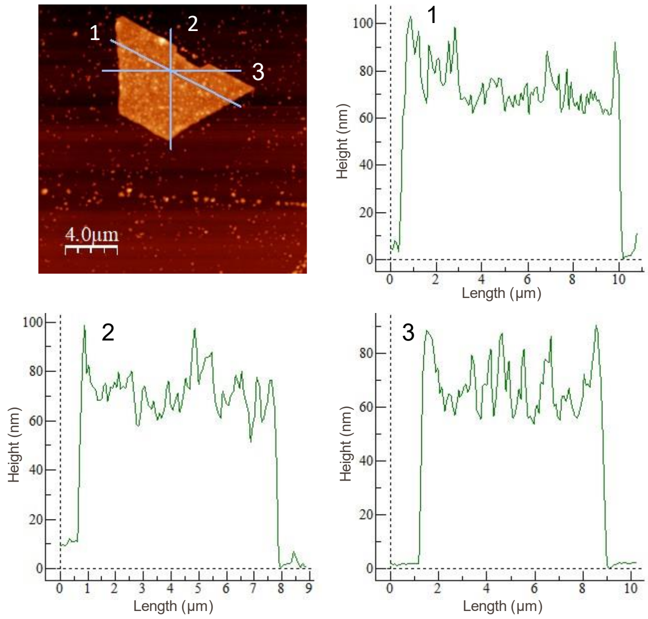

Supplementary Figure 14 Height profiles of a VSe_2 nanosheet prepared by CVD method.

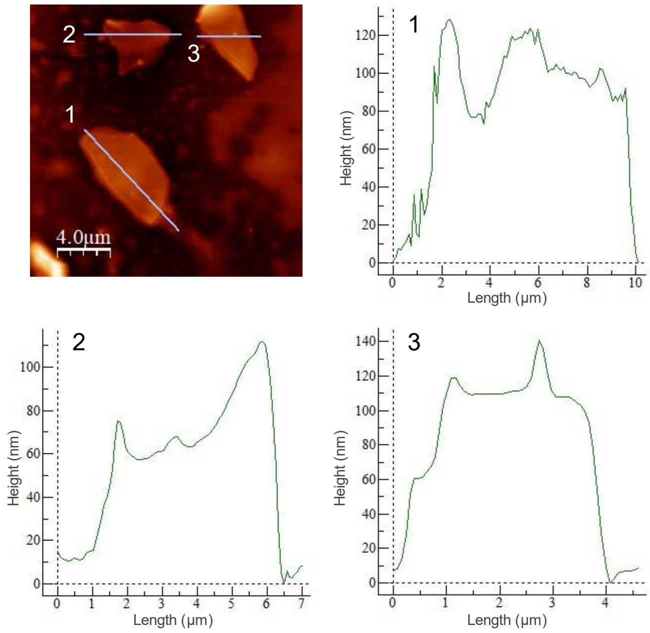

Supplementary Figure 15 Height profiles of $TiSe_2$ nanosheets.

Supplementary Note 10. Heating and thermoelastic wave in VSe_2 on different substrates

As it follows from Supplementary Tables 1 and 2, thermal diffusivity of three substrates differs by one order of magnitude: χ = 0.897 nm^2/ps (quartz glass), 8.385 nm^2/ps (sapphire), and 89.21 nm^2/ps (silicon). It produces a different thermal effect in the VSe_2 nanosheet when the laser pulse is absorbed. Calculated temporal curves of the average temperature increment for a 10-nm VSe_2 nanosheet at the pulse energy of E_p = 12 nJ are shown in Supplementary Fig. 16a. We can see that while an increment in thermal conductivity (silicon) decreases temperature slightly, its decrement (quartz glass) increases it remarkably. However, in the case of an air gap between the nanosheet and the substrate (Supplementary Fig. 16b), the achieved temperature very weakly depends on the properties of the substrate.

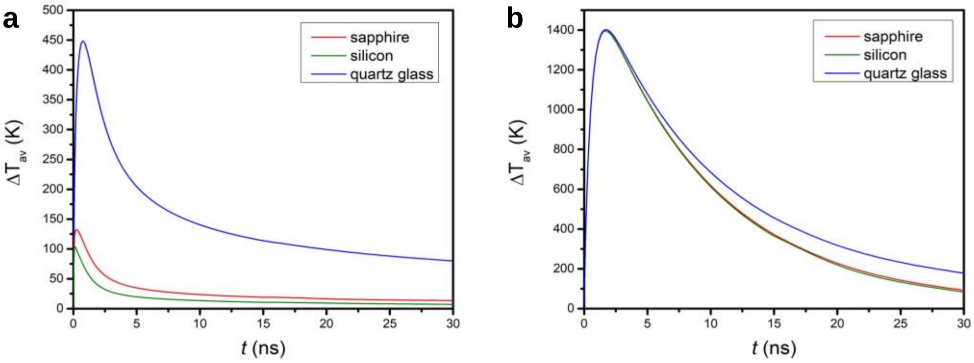

Supplementary Figure 16 Modelling of average temperature increment (ΔT_{av}) of a 10-nm-thick VSe_2 nanosheet on different substrates. a Without gaps. **b** With a 10-nm gap between the nanosheet and the substrate.

This increase of temperature of the nanosheet in the places of a good contact versus unchangeably high temperature of its part with an air gap leads to a decreasing of the thermal stress asymmetry in the case of the quartz glass substrate (Supplementary Fig.

17a), and finally to a lower asymmetry of the momentum of the elastic wave in the nanosheet (Supplementary Fig. 17b).

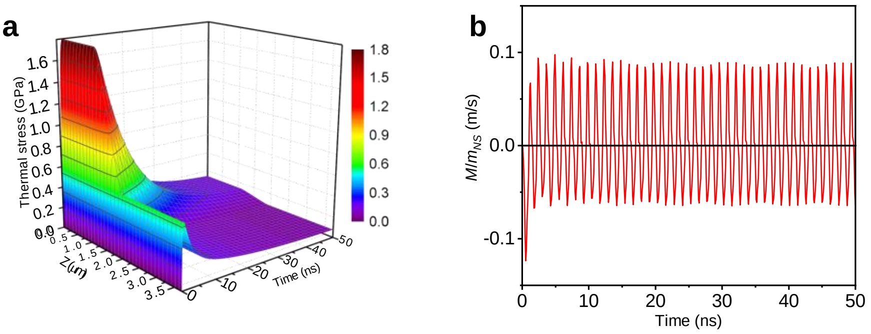

Supplementary Figure 17 Modelling of thermoelastic effect in a 10-nm-thick VSe_2 nanosheet on the quartz glass substrate with a 10-nm edge air gap. a Map of the thermal stress. **b** Momentum-to-mass ratios (M/m_{NS}) dynamics.

Within the model we applied in evaluation of the thermoelastic effect, which does not consider acoustic wave attenuation for simplicity, the wave will propagate until the thermal stress exceeds shear friction stress. Since the latter is several times less in the case of glass substrate, the wave motion will be longer, and despite the lower asymmetry of the momentum, its full integral over time will be comparable with that in case of sapphire. Temporal dependence of the momentum is unchanged after first ~10 ns, and its integral can be characterized by the constant speed value of v_{NS} = 2.8 m/s. Therefore, the nanosheet displacement can be evaluated as $\Delta x \approx v_{NS}t_{\text{end}}$. Assuming friction force on glass 4.5 times less than on sapphire (according to the estimates of the vdW forces in Table 1), an extrapolation of the stress from Supplementary Fig. 17a to longer times, gives $t_{\text{end}} \approx 255$ ns, and $\Delta x \approx 0.7$ nm, which is twice greater than the corresponding estimates we gave in the manuscript for VSe_2 on sapphire ($\Delta x = 0.319$ nm).

Supplementary References

1. Zhang, D. *et al.* Strain Engineering a 4a × √3a Charge Density Wave Phase in Transition Metal Dichalcogenide 1T-VSe_2. *Phys. Rev. Mater.* **1**, 024005 (2017).
2. Israelachvili, J. N. Intermolecular and Surface Forces. (New York, 1992).
3. Maslen, E. N., Streltsov, V. A. & Streltsova, N. R. Synchrotron X-ray Study of the Electron Density in α-Al_2O_3. *Acta Cryst. B* **49**, 973-980 (1993).
4. de Jong, M. *et al.* Charting the complete elastic properties of inorganic crystalline compounds. *Scientific Data* **2**, 150009 (2015).
5. Gordon, J. P. Radiation forces and momenta in dielectric media. *Phys. Rev. A* **8**, 14 (1973).
6. Tamm, I. E. The fundamentals of the theory of electricity, Mir Publishers, Moscow, 1979. Ch.1 (sec. 1.17) & Ch.2 (sec. 2.13).
7. Bayliss, S.C. & Liang, W.Y. Reflectivity and band structure of 1T-VSe_2. *J. Phys. C: Solid State Phys.* **17**, 2193 (1984).
8. Li, L. *et al.* Optical pulse modulators based on layered vanadium diselenide nanosheets. *Nanotechnology* **33**, 065203 (2022).
9. Li, X. *et al.* High-performance vanadium diselenide nanosheets for the realization of compact pulsed fiber lasers. *Ann. Phys.* **533**, 2100230 (2021).
10. Maldonado, M. *et al.* Femtosecond nonlinear refraction of 2D semi-metallic redox exfoliated $ZrTe_2$ at 800 nm. *Appl. Phys. Lett.* **118**, 011101 (2021).
11. Das, A. *et al.* Demonstration of hybrid high-Q hexagonal boron nitride microresonators. *ACS Photonics* **8**, 3027 (2021).

12. Yates, B., Overy, M. J. & Pirgon, O. The anisotropic thermal expansion of boron nitride. *Philos. Mag.* **32**, 847 (1975).

13. Zhou, W. *et al.* Thermal conductivity of boron nitride reinforced polyethylene composites. *Mater. Res. Bull.* **42**, 1863 (2007).

14. Ermolaev, G. A. *et al.* Broadband optical properties of monolayer and bulk MoS_2. *NPJ 2D Mater. Appl.* **4**, 21 (2020).

15. El-Mahalawy, S. H. & Evans, B. L. The thermal expansion of 2H-MoS_2, 2H-$MoSe_2$ and 2H-WSe_2 between 20 and 800 °C. *J. Appl. Cryst.* **9**, 403 (1976).

16. Liu, J., Choi, G.-M. & Cahill, D. G. Measurement of the anisotropic thermal conductivity of molybdenum disulfide by the time-resolved magneto-optic Kerr effect. *J. Appl. Phys.* **116**, 233107 (2014).

17. Gu, H. *et al.* Layer-dependent dielectric and optical properties of centimeter-scale 2D WSe_2: evolution from a single layer to few layers. *Nanoscale* **11**, 22762 (2019).

18. Chiritescu, C. *et al.* Ultralow thermal conductivity in disordered, layered WSe_2 crystals. *Science* **315**, 351 (2007).

19. Ermolaev, G. *et al.* Topological phase singularities in atomically thin high-refractive-index materials. *Nat. Commun.* **13**, 2049 (2022).

20. Moujaes, E. A. & Diery, W. A. Thermoelectric properties of 1T monolayer pristine and Janus Pd dichalcogenides. *J. Phys.: Condens. Matter* **31**, 455502 (2019).

21. Chen, L. *et al.* In-Plane Anisotropic Thermal Conductivity of Low-Symmetry $PdSe_2$. *Sustainability* **13**, 4155 (2021).

22. Buslaps, T., Johnson, R. L. & Jungk, G. Spectroscopic ellipsometry on 1T-$TiSe_2$.

Thin Solid Films **234**, 549 (1993).

23. Leventi-Peetz, A., Krasovskii, E. E. & Schattke, W. Dielectric function and local-field effects of $TiSe_2$. *Phys. Rev. B* **51**, 17965 (1995).
24. Greenaway, D. L. & Nitsche, R. Preparation and optical properties of group IV-VI_2 chalcogenides having the CdI_2 structure. *J. Phys. Chem. Solids.* **26**, 1445 (1965).
25. Wiegers, G.A. Physical properties of first-row transition metal dichalcogenides and their intercalates. *Physica B&C* **99**, 151 (1980).
26. Bhatt, R. *et al.* Thermoelectric performance of Cu intercalated layered $TiSe_2$ above 300 K. *J. Appl. Phys.* **114**, 114509 (2013).
27. Nunez-Regueiro, M., Ayache, C. & Locatelli, M. Thermal conductivity of $NbSe_3$ and $TiSe_2$. *Physica B&C* **108**, 1035 (1981).
28. Bayliss, S.C. & Liang, W.Y. Reflectivity and band structure of 1T-VSe_2. *J. Phys. C: Solid State Phys.* **17**, 2193 (1984).
29. Rugut, E. K. Numerical simulation of structural, electronic and optical properties of transition metal chalcogenides. Dissertation. University of Witwaterstrand, Johannesburg (2017).
30. Wang, S. *et al.* Fabrication and thermoelectric properties of bulk VSe_2 with layered structure. *Solid State Commun.* **318**, 113983 (2020).
31. Yim, W. M. & Paff, R. J. Thermal expansion of AlN, sapphire, and silicon. *Appl. Phys. Lett.* **45**, 1456 (1974).
32. Properties of Sapphire Wafers, Sapphire Thermal Conductivity (valleydesign.com) Valley Design Corp. 2022.

33. Optical materials: IR. Product booklet, Knight Optical Ltd. 2020. IR-material-IR-fused-quartz-JGS3-OPMI-JGS3.pdf (knightoptical.com)

34. Schinke, C. *et al.* Uncertainty analysis for the coefficient of band-to-band absorption of crystalline silicon. *AIP Advances* **5**, 67168 (2015).

35. Haynes, W. M. (ed.) CRC Handbook of Chemistry and Physics, 97th ed., CRC Press (2017).

36. Bolgar, A. S., Trofimova, Zh. A. & Yanaki, A. A. Thermodynamic properties of tungsten diselenide in a broad temperature range. *Soviet Powder Metallurgy and Metal Ceramics* **29**, 382 (1990).

37. Craven, R.A., Di Salvo, F.J. & Hsu, F.S.L. Mechanisms for the 200 K transition in $TiSe_2$: A measurement of the specific heat. *Solid State Commun.* **25**, 39 (1978).

38. Yadav, C. S. & Rastogi, A. K. Electronic transport and specific heat of 1T-VSe_2. *Solid State Commun.* **150**, 648 (2010).

39. Vanadium Selenide | AMERICAN ELEMENTS ® (2022).

40. Park, T. G. *et al.* Interlayer Coupling and Ultrafast Hot Electron Transfer Dynamics in Metallic VSe_2/Graphene van der Waals Heterostructures. *ACS Nano* **15**, 7756 (2021).

41. Ruello, P. & Gusev, V. E. Physical mechanisms of coherent acoustic phonons generation by ultrafast laser action. *Ultrasonics* **56**, 21 (2015).
