# Supplementary material for: Photoacoustic 2D actuator via femtosecond pulsed laser action on van der Waals interfaces
Source: Nat Commun. 2023 Apr 14;14:2135. doi: 10.1038/s41467-023-37763-8 (PMC10104871; doi:10.1038/s41467-023-37763-8)
Supplement: Supplementary file 3 — Description of Additional Supplementary Files [file 41467_2023_37763_MOESM3_ESM.pdf]

Description of Additional Supplementary Files

File name: Supplementary Movie 1

Description: VSe_2 nanosheet movement on the sapphire substrate driven by the femtosecond pulsed laser with the wavelength and repetition frequency of 1040 nm and 1 kHz.

File name: Supplementary Movie 2

Description: A triangular VSe_2 nanosheet movement on the sapphire substrate driven by femtosecond pulsed laser.

File name: Supplementary Movie 3

Description: VSe_2 nanosheet movement driven by the femtosecond laser with the repletion frequency of 1 Hz.

File name: Supplementary Movie 4

Description: All-optical splicing of 2D nanosheets.
